# Supplementary material for: Usability and agreement of the SWIFT-ActiveScreener systematic review support tool: Preliminary evaluation for use in clinical research
Source: PLoS One. 2024 Nov 21;19(11):e0291163. doi: 10.1371/journal.pone.0291163 (PMC11581199; doi:10.1371/journal.pone.0291163)
Supplement: Attachment — Submitted filename: Response to Reviewers.docx [file pone.0291163.s001.docx]

Dear Dr. Marano and Reviewers,

We would like to thank you for your appraisal of our manuscript “*Usability and Accuracy of the SWIFT-ActiveScreener: Preliminary evaluation for use in clinical research”.* We have carefully considered each of the comments provided, and attempted to address each point below. We thank you for giving us the opportunity to revise and improve our manuscript.

We look forward to hearing from you.

**Response to Reviewers**

Editor Revisions

1. Please ensure that your manuscript meets PLOS ONE's style requirements, including those for file naming. The PLOS ONE style templates can be found at:

<https://journals.plos.org/plosone/s/file?id=wjVg/PLOSOne_formatting_sample_main_body.pdf> and <https://journals.plos.org/plosone/s/file?id=ba62/PLOSOne_formatting_sample_title_authors_affiliations.pdf>

Response: We have amended our manuscript to ensure that it follows PLOS ONE’s style requirements provided above.

2. Thank you for stating the following financial disclosure: “This work is supported in part by the Atlas Institute for Veterans and Families”

Please state what role the funders took in the study. If the funders had no role, please state: "The funders had no role in study design, data collection and analysis, decision to publish, or preparation of the manuscript." If this statement is not correct you must amend it as needed.

Please include this amended Role of Funder statement in your cover letter; we will change the online submission form on your behalf.

Response: The funders had no role in this study aside from funding its completion. As such, our financial disclosure statement should be amended to report: “This work is supported in part by the Atlas Institute for Veterans and Families. The funders had no role in study design, data collection and analysis, decision to publish, or preparation of the manuscript.” This has been included in the revised cover letter.

In your revised cover letter, please address the following prompts:

a) If there are ethical or legal restrictions on sharing a de-identified data set, please explain them in detail (e.g., data contain potentially sensitive information, data are owned by a third-party organization, etc.) and who has imposed them (e.g., an ethics committee). Please also provide contact information for a data access committee, ethics committee, or other institutional body to which data requests may be sent.

b) If there are no restrictions, please upload the minimal anonymized data set necessary to replicate your study findings as either Supporting Information files or to a stable, public repository and provide us with the relevant URLs, DOIs, or accession numbers. For a list of acceptable repositories, please see <http://journals.plos.org/plosone/s/data-availability#loc-recommended-repositories>.

We will update your Data Availability statement on your behalf to reflect the information you provide.

Response: We have addressed the above prompts in our revised cover letter and have uploaded a minimal de-identified dataset.

Reviewer One

1. Overall, I think your study is worth publishing but you need to be careful about the claims you make. This an assessment of a potentially valuable tool that benefits from being independent of the tool developers. It would also be useful if you could extract information about how accurate the tool would be with fewer numbers of screeners, and if you could use a well validated method for assessing usability and usefulness.

Response: We thank the reviewer for taking the time to provide feedback. We agree that it would be useful if we were able to extract information regarding the accuracy of the tool with fewer numbers of screeners, and hope to assess this as part of our future work. As such, we have added the following sentence: *“Future directions of this research should consider the assessment of ActiveScreener AI accuracy when including fewer numbers of screeners. Previous research in other areas indicates that ActiveScreener maintains high levels of accuracy with as few as two reviewers [9] and duplication of this result would be beneficial for use in mental health-related systematic reviews with more limited resources.”*

2. Please revise the title of your paper so its clear what Active Screener is.e.g. Usability and Accuracy of the SWIFT-Active Screener tool to support systematic reviews of clinical research

Response: As recommended by the reviewer, we have changed the manuscript title to the following: *“Usability and Accuracy of the SWIFT-ActiveScreener Systematic Review Support Tool: Preliminary evaluation for use in clinical research”*

3. In your Aims section, you justify the study firstly by saying there is little evidence of how Active Screener may perform in evaluations of mental health and treatment outcomes but Howard et al assessed Active Screener on 26 data sets from different fields, what reason is there to believe that mental health field would be different?

Response: While Howard at al. did assess ActiveScreener performance using 26 different datasets, these were primarily studies conducted in genomics, toxicology, and animal models, which may lend themselves to more “straightforward” inclusion and exclusion criteria than the mental health field. Due to the inherent challenges for measurement in psychology (please see Osborne 2010 *“Challenges for quantitative psychology and measurement in the 21^st^ century”),* and that the mental health field continues to update its diagnostic criteria for numerous diagnoses, the creation of inclusion/exclusion criteria, and training reviewers on said criteria, may become more complicated. We felt that due to these challenges it was important to discern whether the ActiveScreener AI was able to accurately predict which articles should and should not be included during the initial phase of screening when conducting a mental-health focused review. We have added more information in the manuscript to clarify this.

4. The description of the evaluation is not detailed enough for it to be replicated. Some description of how Active Screener works is needed (particularly as it has two facets, i. ordering articles in terms of specified criteria and ii. identifying when a certain percentage of expected relevant papers have been found), and then you need to explain how the 18 participants interacted with the system. Did every screener review each article in the same order, or did screeners who accessed the system at different times see different articles? Did the screeners get any feedback on their performance? You report that your results are better than the results reported by the tool developers, but is this because you have 18 screeners? Would the results be closer to Howard et al.'s results, if you had only two reviewers? In your Aims section you also justify your study by saying " Further, the precision of the estimation model remains unclear", however, Howard et al's estimate of precision was based on the model of the screening process they used in their evaluation. If you found better precision, this could be due to your evaluation process being based on 18 screeners.

Response: We appreciate the feedback that our description was not specific enough, and as such, have cited Howard et al. description of the ActiveScreener software with the following additions: *“Once records have been imported into ActiveScreener, the software uses topic modeling, which is the clustering of related documents to discover computationally derived common themes, which are then used in combination with the training set of data [8]. In this case, the “training set” is the real time manual identification of uploaded documents relevant or not relevant. Based on user feedback via patterns of screening, ActiveScreener uses its pretrained algorithm to build a log-linear model estimating the probability that an article should be included versus excluded. This AI prioritization of articles is believed to be most relevant during screening, and can trim screening time and human effort by nearly 70% [8]”*

We have further clarified the role of the screeners and how they accessed ActiveScreener on their own time in the Part 1 procedure. This now reads “*A total of 10002 references required review at the title and abstract stage. ActiveScreener accuracy statistics were set at 95% resulting in 5390 of these references to be reviewed in duplicate by screeners. Screeners were able to access ActiveScreener at any time on their own schedules, and when they logged on, would be provided with the most relevant article at that time as identified by ActiveScreener rather than every screener reviewing articles in the same order.”*

We agree that a higher number of screeners may have partially led to the increased accuracy of the prediction software as suggested by the reviewer. While we believe that the high accuracy of ActiveScreener would have been maintained with a lower number of screeners, this determination was not one of our objectives and as such, we have added the following to the beginning of our Discussion: *“While the software was expected to reach 95% accuracy, the actual accuracy of its machine learning model in our review exceeded 95% (97.9%), which may have been aided by the high number of independent screeners on this project.”*

5. For assessing personal opinions you would have better to have used one of the well known technology evaluation methods, for example the Technology Acceptance Model (TAM) which uses a number of questions (similar to those you used) to assess the usability and usefulness of a technology. It is much better to used a previously validated model than invent you own set of likert-format ordinal scales. Also, you should be precise about the nature of questions, you seem to be equating an open question with a free text response as qualitative data, and calling any closed questions quantitative data, even those referring to subjective opinions based on a likert-format agreement scale. Another advantage of the TAM is that their is a process for aggregating the collected data into an overall assessment of the subjective factors. See any of the many papers discussing the model such as: Marangunić, N., Granić, A. Technology acceptance model: a literature review from 1986 to 2013. Univ Access Inf Soc 14, 81–95 (2015). https://doi.org/10.1007/s10209-014-0348-1.

Richard J. Holden, Ben-Tzion Karsh, The Technology Acceptance Model: Its past and its future in health care, Journal of Biomedical Informatics, Volume 43, Issue 1, 2010, Pages 159-172

Response: We appreciate the reviewer’s detailed comments regarding the use of an already validated tool rather than designing our own survey to assess usability and experiences with ActiveScreener. While TAM is an excellent measure for acceptability of technologies, we made the decision to create our own survey to assess the explicit experiences with ActiveScreener, as well as the training that was provided to learn how to use this software. The TAM is meant for a wide range of technologies and as such, did not have the degree of specificity that we hoped to obtain in responses (e.g., regarding whether there were glitches in the software).

We appreciate the reviewer’s comment regarding qualitative vs. quantitative data. We decided to use the term qualitative data to describe the open text boxes, as we thematically coded the responses. As this is an interpretation-based, descriptive analysis of the text responses provided by screeners, we felt it was most in-line with using the term “qualitative” to describe the approach.

Reviewer Two

This is an interesting and useful paper. I have outlined my queries and comments below (in the order they appear in the manuscript). The most important of which is point 3. I hope you find them helpful.

1. The flow of the introduction could be improved. For example: You provide a statement about the aim of the paper in the middle of the introduction (page 3, sentence beginning "In this paper...") and repeat it later on. I suggest removing this.

Response: We have removed the stated aim from earlier in the introduction as recommended.

2. You introduce ActiveScreener and it's purpose of reducing screening time (page 3, final sentence of 1st paragraph), then discuss the process of conducting reviews and resource issue (page 3, paragraph 2), then introduce software to support review (page 4, paragraph 1). It would make more sense to me discuss the process of conducting reviews and resource issues then introduce software more generally then talk specifically about ActiveScreener.

Response: We appreciate the reviewer’s comment regarding improving the flow of our paper. As suggested, we have removed the initial introduction to ActiverScreener on page 1.

3. I would be interested to know why you chose ActiveScreener over other software. Does it offer something different to other tools?

Response: We chose ActiveScreener over other software options to due its ability to classify and predict whether an article should be included, while also providing users with information regarding predictive screening accuracy. This software specifically provides the opportunity to reduce time spent on initial abstract and title screening, which can be arduous depending on how wide or narrow the developed inclusion criteria are. In order to clarify this point, we have the following to the introduction:*“In this paper, we evaluated ActiveScreener in terms of its accuracy and usability in a large SR of mental health outcomes following treatment for PTSD. ActiveScreener was selected for this project namely for its departure from programs that uses AI to identify records, and instead, uses machine learning to build a predictive algorithm to reduce time spent in screening phases of SRs.”*

4. The description of how the 'accuracy' part of the paper was conducted is not clear to me. For example, what does it mean to set the accuracy of ActiveScreener to 95? Was ActiveScreener trained using the initial 5390 references, then the remaining 4612 references were assessed by both ActiveScreener and human readers? Does assessment happen independently, or do the human readers see ActiveScreener inclusion/exclusion decisions? Perhaps it could be presented as a flow diagram?

Response: Thank you for your comment. The algorithm that ActiveScreener uses was set to 95% accuracy, which meant that in order to reach the threshold of 95% of studies that “should” be included, being included in the full-text screening, 5390 references had to be reviewed by the systematic review screeners. ActiveScreener is trained on an ongoing basis, based on the dataset, and following which types of articles are included/excluded during this initial phase. When the accuracy statistics were reset to 100%, this was the way to tell the ActiveScreener algorithm that every single paper which “should” be included in the review needed to be captured, leading to the screening of the remaining 4612 references. Human screeners do not have access to the individual decisions of ActiveScreener as they are screening, but can see a graph visualization of how “close” the screening has gotten to including 95% of the appropriate articles. In order to clarify how this software works, we have added text at the end of the introduction and in the Part 1 procedure.

5. Throughout the paper you refer to the 'accuracy' of assessment. Accuracy implies that inclusion/exclusion decisions are being compared against the truth (i.e. that a paper should be included in the review because it meets the inclusion criteria at the point of full text assessment). But this isn't what is being assessed with the method employed. It is agreement between ActiveScreener and the human readers on decisions at title/abstract assessment. You could change 'accuracy' to 'agreement' throughout. But this doesn't tell us anything about whether ActiveScreener is categorising papers appropriately (which is what I, as a reviewer, need it to do). You need to compare decisions made at title/abstract to final decisions at full text assessment to do this.

Response: Thank you for your comment. We have changed accuracy to agreement where appropriate. Agreement was high between screeners and ActiveScreener for the first phase of screening (title and abstract), and as the confusion matrix assessed the accuracy of decisions by ActiveScreener compared to human screeners (human screener determinations of inclusion/exclusion were considered the “correct” result at this stage, as all abstracts that were included by human screeners were then assessed at the full-text phase), the term accuracy has been maintained in certain contexts. Agreement was also measured using Kappa, and has been included in the manuscript. As there is currently no option for full-text assessments by ActiveScreener AI algorthim, we are unable to determine the accuracy or agreement for final inclusion of full-texts. However, as this initial algorithm could be further developed for a full-text article screening, we felt that it was important to assess the initial algorithm’s predictive accuracy for the first stage of screening in isolation.

6. Agreement is present between ActiveScreener and the whole cohort of human screeners. It would be useful to have this information by human experience so we can see if the tool is more/less helpful for different levels of experience. Especially as half of the samples has not conducted a review before.

Response: We agree that it would be helpful to know whether more experienced reviewers would lead to further heightened accuracy of ActiveScreener with less articles reviewed. However, due to the large scope of this review, we were unable to use only one experience level of screener (i.e., only expert or novice). Moving forward, this would be an important contribution to the literature. As such, we have added the following in our discussion: *“Future directions of this research should consider the assessment of ActiveScreener AI accuracy when including fewer numbers of screeners and with different levels of experience. Previous research in other areas indicates that ActiveScreener maintains high levels of accuracy with as few as two reviewers [9] and duplication of this result would be beneficial for use in mental health-related systematic reviews with more limited resources.”*

7. Do you have any extra information about the 97 papers for which there was no agreement between ActiveScreener and the human screeners? Were the human readers correct to include these papers? Was there something different about them that made it hard for ActiveScreener to interpret them?

Response: The 97 articles that were not in agreement were identified by the ActiveScreener AI as conflicted. In this situation, the AI categorized that humans would have a difficult time agreeing on whether to include these studies or not due to the language that was used in the manuscripts. The human readers were correct in including these papers for full-text screening, as they still did contain keywords of relevance, however, based on the algorithm used by ActiveScreener AI, the articles contained fewer keywords that were “typically” used in other, previously included articles.

8. Please can you provide some description of how the survey was developed. Has it been assessed for reliability and validity? If yes, what are they? If not, why not? What are the implications for using a survey that has not been assessed for reliability/validity (to add to the discussion, where appropriate).

Response: We appreciate the concern with regards to measurement validation. As this was an internal project to assess usability and acceptability of the software, we did not assess reliability of the constructs, and only examined face validity of the measure. We decided not to assess reliability and validity in depth due to the limited use of this survey measure external to this context. Due to this, we have added the following at the end of the discussion: *“Of note, while the administered survey was developed internally and assessed for face validity, reliability and other forms of validity were not examined. As such, this may have led to measurement error and quantitative results should be interpreted with caution.”*

9. The discussion states ActiveScreener can reduce screening time and effort (page 17, paragraph). Where in the methods does it explain how amount of screening time and effort are assessed? And where in the results does it report the results of these?

Response: There was no explicit measure of the amount of screening time saved in this project, as all 10002 articles ended up being screened by screeners. As such, there is no description of time reduction assessment. However, anecdotal evidence suggests that this screening process would have taken nearly half the time if we had employed the ActiveScreener “stop points”, considering that when the threshold of 95% accuracy was reached, our screeners had screened just over half of the uploaded articles. Assuming that the amount of time spent screening would be maintained in an approximately equal fashion between the first half and the last half of the uploaded article dataset, it stands to reason that when used appropriately, ActiveScreener can save researchers time and human resources when conducting systematic reviews. We have added expanded on this consideration at the beginning of the discussion.
