# Supplementary material for: Usability and agreement of the SWIFT-ActiveScreener systematic review support tool: Preliminary evaluation for use in clinical research
Source: PLoS One. 2024 Nov 21;19(11):e0291163. doi: 10.1371/journal.pone.0291163 (PMC11581199; doi:10.1371/journal.pone.0291163)
Supplement: Attachment — Submitted filename: Response to Reviewers.docx [file pone.0291163.s002.docx]

Dear Dr. Marano and Reviewers,

We would like to thank you for your further appraisal of our manuscript titled “*Usability and Accuracy of the SWIFT-ActiveScreener: Preliminary evaluation for use in clinical research”.* We have attempted to address each new point provided by reviewers for consideration below. We thank you for giving us another opportunity to revise and improve our manuscript.

We look forward to hearing from you.

**Response to Reviewers**

Reviewer Two

Thank you for your considered responses and revisions to my review.

Response: We appreciate the time the reviewer took to provide feedback to our manuscript, and have attempted to address comments below.

1. My remaining issue is with accuracy/agreement. If the human reviewers and ActiveScreener are both terrible at identifying relevant literature (e.g. they miss 99% of studies that would be included at full text assessment) they might well have excellent ‘agreement’. But they are not ‘accurate’. Agreement with poor performance would introduce more problems than it would solve. Unfortunately, the study design employed here doesn’t enable us to understand the impact that ActiveScreener would have on the review process. Without being able to compare the title/abstract decisions to the final inclusion/exclusion decisions, the conclusions about the usefulness of ActiveScreener should be tempered.

Response: We thank the reviewer for taking the time to provide further feedback for the improvement of this manuscript. We acknowledge that agreement with poor performance would be problematic and introduce a host of negative issues when attempting to use a tool such as this, and have tried to implement your suggestions throughout.

2. Make it clear in the methods section that what is being examined is the agreement between ActiveScreener and the reviewers’ decisions at title/abstract screening.

Response: As recommended by the reviewer, included the following sentence in the methods section *“Screening results for the remaining 4612 references from ActiveScreener and the screeners were then compared to assess agreement between ActiveScreener and reviewer’s decisions during title/abstract screening.”*

3. Use the term ‘agreement’ throughout.

Response: We have attempted to use the term agreement rather than accuracy throughout the manuscript as the request of the reviewer.

4. Acknowledge that because the decisions at title/abstract assessment are not compared against final inclusion decisions, it’s not possible to tell what impact ActiveScreener would have on the review process.

Response: We have included a sentence in the discussion section acknowledging that the final impact of ActiveScreener is not captured in this paper. This sentence reads: *“Importantly, as decisions at the title and abstract phase were not compared directly against final inclusion decisions in this analysis, it is not clear the magnitude of impact ActiveScreener has on the screener process in its entirety. However, one can assume with relative confidence that due to the high agreement in phase one, high levels of agreement would have been maintained.”*

Reviewer Three

This article is well-written and it is clear that a lot of work was done to evaluate the tool. Especially the high number of screeners that used the tool contributes to the evaluation's quality. My comments are mostly about the clarity of descriptions of how the tool works, and what the AI predicts.

Response: We would like to thank the reviewer for this comment, and have attempted to address further comments below.

1. I do agree with reviewer 1 comment 4, where they mentioned that the description of how ActiveScreener works needs to be more clear. In the updated manuscript you added this sentence: 'the software uses topic modeling, which is the clustering of related documents to discover computationally derived common themes, which are then used in combination with the training set of data [8].' but to the best of my knowledge this statement is incorrect according to the description provided by Howard et al. (2020), who explicitly mention a L2-regularized log-linear model. They do refer to an older paper which describes a different software that, among others, uses topic modelling but they do not mention any topic models as part of ActiveScreener. Howard et al (2020) also do not mentioned that ActiveScreener uses a pre-trained model, they do in fact describe active-learning which means that the model is trained from scratch for every new review project. You are correct to point out that the model is a log-linear model, but this is used to re-order the references as they are screened, so the screeners are always presented with the most likely relevant references. This first algorithm does not predict if an article is relevant or irrelevant, it sorts them by likely relevancy. A potential description for this model could be along the lines of 'ActiveScreener uses a L2-regularized log-linear model and active-learning approach. This means that the AI is continually trained during the screening process; whenever screeners identify included articles the model is re-trained and orders likely relevant references to the top of the screening pile'.

Response: We appreciate the time the reviewer has taken to address this statement and ensure the accuracy of our ActiveScreener description, as the description that we used was inadvertently out of date. We have incorporated the reviewer’s suggested changes to the description of how ActiveScreener works. This now reads *“ActiveScreener uses a L2-regularized log-linear model and active-learning approach to screening, meaning that the model is continually trained during the screening process [8]. In this case, the “training” occurs whenever a screener identifies included articles through the real time manual identification of uploaded documents. Each time an article is identified as “included” the model is re-trained and re-orders the most likely relevant references to be screened next.”*

2. What remains unclear in the revised manuscript is that the system combines two different AI approaches: firstly the active learning (ie. re-ordering of references during screeing with the log-linear model described above) and secondly the statistical algorithm predicting the 95% inclusion rate, as described by Howard et. al (2020). It is this second algorithm that tells screeners when to stop, and thus causes the time-savings. It might be beneficial if this distinction was clear in the manuscript, as previously noted by the other reviewer as well. For the second model, a description could be along the lines of 'Sensitivity within the screening process is then estimated using a statistical model based on a negative binomial distribution. This model alerts screeners when a certain threshold, for example 95% of all likely relevant articles, have been included (Howard et al. 2020)

Response: We agree that it would be beneficial for readers to understand that the system itself uses two separate AI approaches to re-order likely relevant articles and generate sensitivity estimates. We have therefore included the following sentence in the description of the tool, which is based on the reviewer’s feedback: *“A statistical model based on a negative binomial distribution is then used by ActiveScreener to estimate sensitivity of the screening process, and is used to alert screeners that a specified threshold of likely relevant articles (in this case, 95%) have been included.”*

3. This sentence “While the software was expected to reach 95% accuracy, the actual accuracy of its machine learning model in our review exceeded 95% (97.9%), which may have been aided by the high number of independent screeners on this project.” ActiveScreener predicts the inclusion rate of relevant articles, in other words: the sensitivity (and not accuracy). It might be a good idea to replace 'accuracy' with 'sensitivity' in this sentence if I don't misunderstand your evaluation. Sensitivity is the fraction of included references that were found during screening, ie. 95% sensitivity means that 95% of includes were found at the time when the tool told screeners to stop. Or in your case, 97.9% were actually found, which shows that the tool was conservative and its prediction on the safe side, as also described by Howard et. al (2020) with their 26 evaluation reviews. Since you are also evaluating other aspects of the system and the way how the log-linear model performs it might be worth thinking about using the term 'reliability', for example in the title. Could you please consider reviewing the use of the word 'accuracy' in all contexts and consider using 'sensitivity', or 'reliability' or 'agreement' whenever you did not explicitly calculate the metric known as 'accuracy'?

Response: Throughout the text, we have replaced the term accuracy with either agreement or sensitivity in order to reduce confusion and ensure clarity of our processes.

4. Line 115 please consider using 'ActiveScreener inclusion statistics were set at 95% predicted sensitivity [..]' or 'predicted inclusion rate' for clarity.

Response: We have added the term “predicted inclusion rate” in order to improve clarity of this sentence as requested.

5. It is unclear to me how ActiveScreener predicted a reference as conflicted? Do you mean that there was an unresolved screening conflict between reviewers and that the tool pointed out that there were conflicts?

Response: ActiveScreener only identified a reference as conflicted if two human screeners disagreed on whether the reference should be included or excluded. When we requested reviewers to stop screening in order to assess agreement, there were unresolved conflicts which were captured as conflicted. As such, the confusion matrix predictive agreement included a class for conflicted references. This was done to see if ActiveScreener would be able to predict with good agreement, articles which would be difficult for human screeners to decide whether to include or exclude. In this case, we found that the human screeners included 97 articles which the confusion matrix had identified as conflicted.

6. I do agree with your response about the TAM and that there was indeed value in explicitly evaluating the tool.

Response: Thank you for your comment. We will be sure to consider the use of the TAM in future research where appropriate, as it is a useful evaluation tool.
