# Supplementary material for: Usability and agreement of the SWIFT-ActiveScreener systematic review support tool: Preliminary evaluation for use in clinical research
Source: PLoS One. 2024 Nov 21;19(11):e0291163. doi: 10.1371/journal.pone.0291163 (PMC11581199; doi:10.1371/journal.pone.0291163)
Supplement: Attachment — Submitted filename: PLOS ONE - Response to Reviewers.docx [file pone.0291163.s003.docx]

Thank you for reconsidering our manuscript titled "*Usability and agreement of the SWIFT-ActiveScreener systematic review support tool: Preliminary evaluation for use in clinical research*". We have thoroughly addressed all remaining comments and suggestions.

**Response to Reviewers**

**Reviewer #2:**

Thank you for your helpful revisions. A couple of final remarks.

*Response: We thank the reviewer for taking the time to provide further feedback for the improvement of this manuscript.*

1. There are a few places where it would be helpful to be more explicit about what is being done by adding “at the title/abstract assessment phase of the review” at the end of each sentence, i.e.

- Line 40 - inclusion or exclusion of relevant articles “at the title/abstract assessment phase of the review”.
- Line 107 - Eighteen screeners were trained to identify articles for inclusion and exclusion “at the title/abstract assessment phase of the review”.
- Line 239 - In our study, we found that ActiveScreener performed above its expected 95% agreement in prediction “at the title/abstract assessment phase of the review”

*Response: We have added the requested phrases into the manuscript as suggested.*

2. There’s a sentence in the abstract and conclusion (lines 43 and 309) that I don’t think accurately reflects the state of knowledge regarding ActiveScreener:

- Our results showed that ActiveScreener, when used appropriately, may save considerable time and human resources when performing SR

*Response: We appreciate the reviewer’s comment and have adjusted these sentences accordingly.*

In the absence of evidence about whether the title/abstract decisions were correct, we cannot tell what impact ActiveScreener will have on the review process. We only know that it performed similarly to the sample of human reviewers at this stage of the review.

*Response: Thank you for your comment. We have noted this as a limitation within our discussion section.*

**Reviewer #3:**

Thank you for addressing the comments in detail, the paper looks good now. I recommended it for publication.

*Response: Thank you for dedicating your time to review our manuscript. Your insightful feedback was immensely appreciated and will undoubtedly contribute to enhancing the quality and impact of this manuscript.*
