# Supplementary material for: Usability and agreement of the SWIFT-ActiveScreener systematic review support tool: Preliminary evaluation for use in clinical research
Source: PLoS One. 2024 Nov 21;19(11):e0291163. doi: 10.1371/journal.pone.0291163 (PMC11581199; doi:10.1371/journal.pone.0291163)
Supplement: Attachment — Submitted filename: Response to Reviewers.docx [file pone.0291163.s004.docx]

Thank you for reconsidering our manuscript titled "Usability and agreement of the SWIFT-ActiveScreener systematic review support tool Preliminary evaluation for use in clinical research". We have thoroughly addressed all remaining comments and suggestions below and in the manuscript.

# Response to Reviewers

# Reviewer #3: (no response needed)

I am sorry for missing this tiny detail in my previous review. You revised the description of ActiveScreener's machine-learning according to my previous comments but I just realised one likely incorrect sentence remained: page 4 line 84 about the pretrained model. ActiveScreener uses active learning, and NOT a pretrained model. This was described by Howard et. al in your cited reference in section 2.2, bag-of-words and tf-idf is used to represent text and then a L2-regularized log-linear model and active-learning follows. As someone who works with these models on a daily basis, I can tell you that there is an important difference between pretrained and tf-idf initialised models.

I believe there are two ways forward: could you please delete the sentence about the pretrained model that isn't supported by the cited literature; or if you would like to keep it, could you please contact Sciome and confirm that a pretrained model is actually used? If you do decide to contact them, I found their email address: swift-activescreener@sciome.com

*Response: We appreciate your attention to detail in order to improve our paper. As requested, we have deleted the sentence (lines 88-89).*

# Reviewer #4: (response required)

The authors assessed the agreement and usability of the ActiveScreener in the large SR on mental health outcomes following treatment for PTSD. However, there are several issues that need to be addressed before this study can be considered for publication.

In the 'Introduction' section, the authors should focus on reviewing current methods for conducting SRs and in particular compare their disadvantages and advantages.

*Response: We appreciate the reviewer’s feedback and have provided references throughout the introduction to protocols which provide “gold standard” systematic review methodologies for readers to refer to when conducting a systematic review. We have included the following sentences: “Standardized protocols, such as the Preferred Reporting Items for Systematic Reviews and Meta-Analysis (PRISMA), offer researchers a roadmap to conducting SRs with rigour and fidelity [3]. In addition, formal guides established by Cochrane provide further evaluation criteria in order to provide appropriate context for the interpretation of study data in various research settings [4].” (lines 54-58)*

*Further, we have included the typical methodology for screening articles for inclusion while conducting a systematic review on lines 64-70. As the software we reviewed primarily reduced the workload during the screening process, we felt it was prudent to focus on this component of a systematic review rather than reviewing the entire SR process, which is outside the scope of this manuscript.*

There are so many sections that it becomes confusing and unreadable, the authors should re-integrate the whole article, e.g. integrate the methods and results of Part I and Part II separately.

*Response: We have integrated the methods and results as requested by the reviewer.*

Authors need to pay attention to the formatting of the article, pauses between paragraphs, spaces at the beginning of paragraphs, etc.

*Response: Thank you for your recommendation. We have thoroughly reviewed and corrected the formatting issues, ensuring proper pauses between paragraphs and consistent spacing at the beginning of each paragraph. We believe these changes improve the overall readability and presentation of the article.*

What is innovative about the article? What are the advantages and disadvantages of ActiveScreener compared to other tools?

*Response: This innovation of this article is due to the fact that it is the first manuscript to assess the utility of ActiveScreener using a large scale, mental health focused systematic review. Over ten thousand references required review during the title and abstract screening phase of this project, leading to a large number of articles from which to compare agreement between human screeners and the ActiveScreener algorithm. Other previous articles which have reviewed ActiveScreener’s utility have assessed it using smaller numbers of articles and different research areas.*

*For additional reader clarification, we have also included information regarding the novelty of ActiveScreener compared to other, currently used systematic review tools such as Covidence on lines 77-86, which read: “For example, COVIDENCE aids clinical research reviews with its ability to distinguish between randomized controlled trials (RCTs) versus non-RCTs. Other systematic review tools include similar options to apply sorting tags and take notes. Critically, ActiveScreener is a novel SR tool due to its use of active learning, which allows the software to estimate completeness of the screening process, and notifies reviewers when they may stop screening early. In this paper, we evaluated ActiveScreener in terms of its agreement with human screeners, and usability in a large SR of mental health outcomes following treatment for PTSD. ActiveScreener was selected for this project namely for its departure from programs that use AI to identify records, and instead, use machine learning to build a predictive algorithm to reduce time spent in screening phases of SRs.”*

This manuscript requires careful editing and special attention to English grammar, spelling and sentence structure.

*Response: We appreciate your feedback and have made adjustments to grammar, spelling, and sentence structure throughout the manuscript in consideration of this concern.*

# Reviewer #5:

Thank you for addressing the comments in detail, The paper looks good. I recommended it for publication.

*Response: Thank you for your positive feedback and recommendation for publication. We're thrilled to hear that the revisions have met your expectations and look forward to sharing our work with a wider audience.*
