# Supplementary material for: Usability and agreement of the SWIFT-ActiveScreener systematic review support tool: Preliminary evaluation for use in clinical research
Source: PLoS One. 2024 Nov 21;19(11):e0291163. doi: 10.1371/journal.pone.0291163 (PMC11581199; doi:10.1371/journal.pone.0291163)
Supplement: Attachment — Submitted filename: Response to Reviewers.docx [file pone.0291163.s005.docx]

**Response to Reviewers**

**Reviewer #6**

*I have no further review comments to authorship team at this point. This has undergone extensive peer review already*

- **Author Response:** Thank you for your feedback and for confirming that no further comments are needed at this stage. We appreciate the time and effort dedicated to the extensive peer review process and are pleased that the revisions have met the necessary standards.

**Reviewer #7**

*Thank you for the opportunity to review this manuscript. I am happy to see more literature on the effectiveness of machine learning to increase screening efficiency in the title and abstract phase of systematic reviews (SRs), given the importance of not missing relevant records when performing this step. I have a couple of follow-up questions pertaining to question 4 above (full availability of underlying data) and about the ethics statement response provided with the manuscript, which indicated that an ethics statement was not applicable: I may have missed it, but I could not see the supporting information files with the underlying data. However, I admit to not being entirely familiar with the particular requirements for the granularity of the data and I answered "yes" based on the statement provided by the authors.*

- **Author Response:** The authors would like to thank the reviewer for their time and efforts providing a comprehensive review of this paper. To clarify the question raised with respect to the availability of underlying data, we included all available raw data in Table 1 (ActiveScreener data) and Table 2 (survey data) within the manuscript.

*I was also wondering if it would be pertinent to explain why ethics review was not needed, given the involvement of human participants in the survey component in particular. Would this be considered a quality improvement project?*

- **Author Response:** Yes, because this survey was administered as a normal operational requirement for quality assurance and improvement, this survey would be considered a quality improvement project and would not require REB review per the Tri-Council Interagency Advisory Panel on Research Ethics (TCPS 2, 2022; Chapter 2, Article 2.5).

*There are a few small comments I have regarding some of the information provided in the manuscript. Line 56: PRISMA is a reporting guideline rather than a guide to conducting SRs: I would suggest adjusting the wording accordingly, per reference 3 cited, which states "PRISMA 2020 is not intended to guide systematic review conduct, for which comprehensive resources are available." See also* [*https://link.springer.com/article/10.1186/s13643-021-01671-z*](https://link.springer.com/article/10.1186/s13643-021-01671-z) *for an editorial on this point.*

- **Author Response:** We have adjusted the wording to better reflect the nature of the reporting guidelines (see highlighted p.3, ll. 54-57)

*Line 79-80: I would suggest rewording this phrase a little, e.g., to "ActiveScreener is part of a growing set of novel tools that use active learning..." - Covidence includes this functionality since 2022 (see* [*https://www.covidence.org/blog/release-notes-december-2022-machine-learning/*](https://www.covidence.org/blog/release-notes-december-2022-machine-learning/)*) and other products have been using active learning for several years now as well (DistillerSR AI, EPPI-Reviewr to name a couple). If ActiveScreener is novel in a particular way, it would be worth emphasizing how (for example, are there other screening tools using active learning that notify reviewers when they may stop screening? Is it novel in that particular way?)*

- **Author Response:** We agree and made the appropriate edits (see highlighted p.4, ll. 83-86)

*I found the manuscript to be clear and effective at communicating the results.*

- **Author Response:** Thank you for this comment.

*Thanks again for providing this information about ActiveScreener, which will hopefully soon be available for other researchers to use to support their own decisions about how to undertake the screening process more efficiently and feasibly.*

- **Author Response:** Thank you for this comment.
